# Supplementary material for: The Harvest suite for rapid core-genome alignment and visualization of thousands of intraspecific microbial genomes
Source: Genome Biol. 2014 Nov 19;15:524. doi: 10.1186/s13059-014-0524-x (PMC4262987; doi:10.1186/s13059-014-0524-x)
Supplement: Supplementary file 1 — SNPs unique to each method characterized by the five most common splits found. Figure S1. Runtime comparison for the whole-genome alignment methods on the simulated 32-genome E. coli W3110 dataset. Figure S2. Timing performance from 32 to 10,000 S. pneumoniae genomes. [file 13059_2014_524_MOESM1_ESM.docx]

**Supplementary Materials**

- **Supplementary Table 1**. SNPs unique to each method characterized by the five most common splits found.
- **Supplementary Figure 1**. Runtime comparison for the whole-genome alignment methods on the simulated 32-genome *E. coli* W3110 dataset.
- **Supplementary Figure 2**. Timing performance from 32 to 10,000 *S. pneumoniae* genomes.

**Supplementary Table 1**. SNPs unique to each method characterized by the five most common splits represented. Combined, these five splits account for approximately half of the unique SNPs and ~70% of the unique SNP positions. **Col Cnt:** the number of alignment columns (i.e. SNP positions) supporting the split**. Split:** The identified split overlaid on the reference tree, with the highlighted genomes forming one half of the split. **SNP count:** the total number of SNPs that pertain to the split.

| Method | Col Cnt | Split | SNP count |
| --- | --- | --- | --- |
| Parsnp | 28 (1%) | 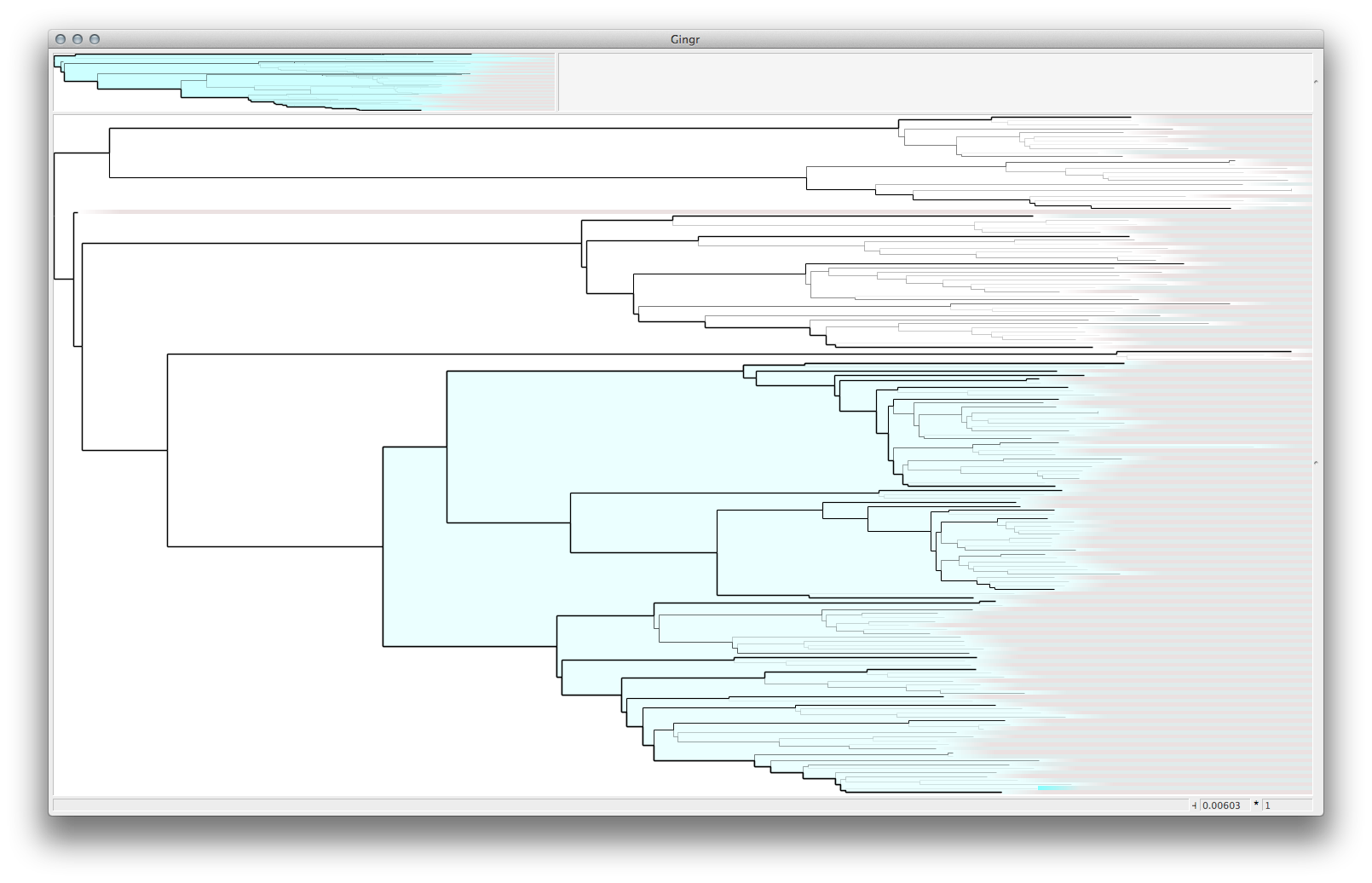 | 3052 (21%) |
| Parsnp | 129 (6%) | 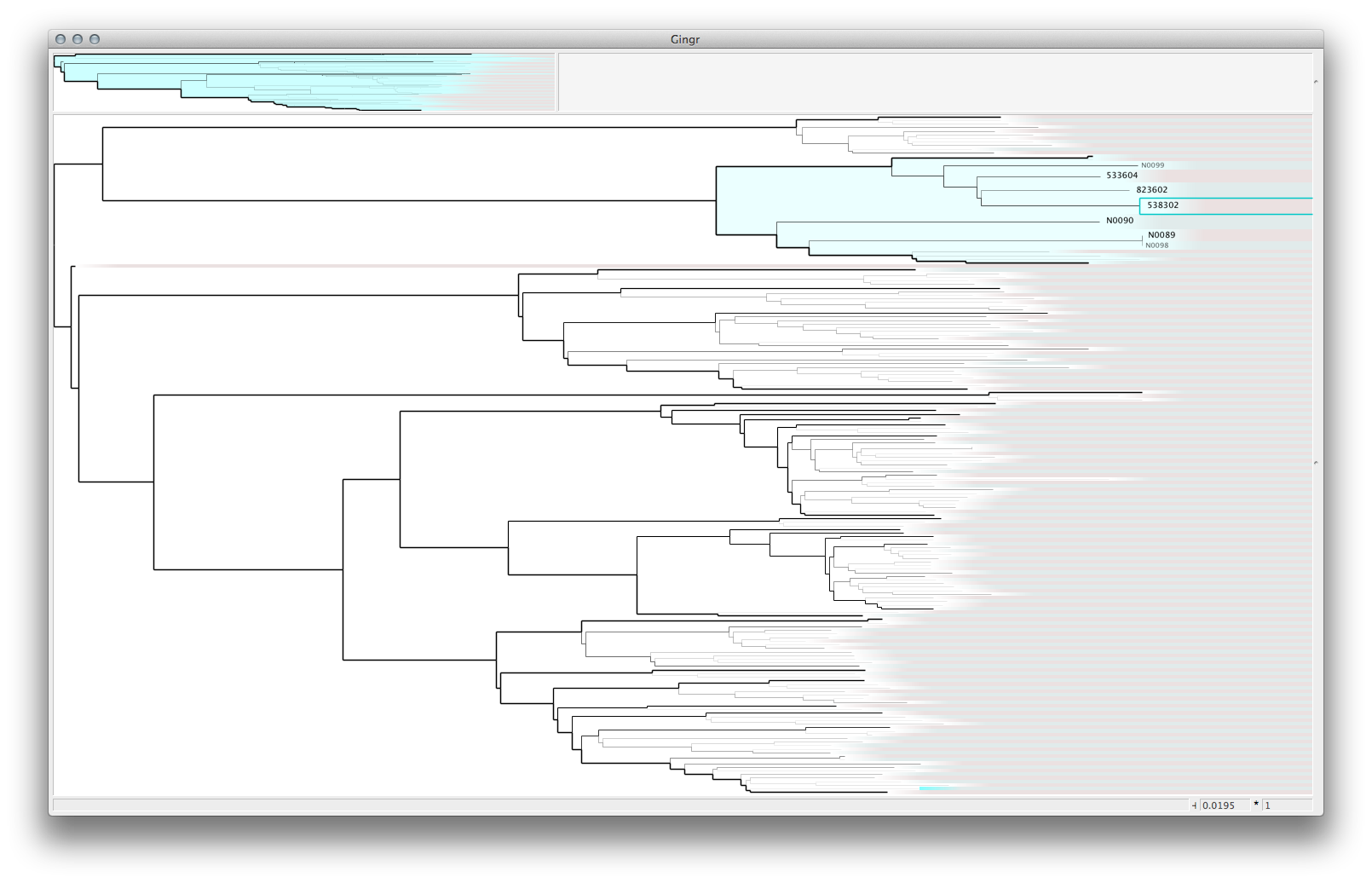 | 1677 (11%) |
| Parsnp | 1503 (66%) | Single genome splits | 1503 (10%) |
| Parsnp | 23 (1%) | 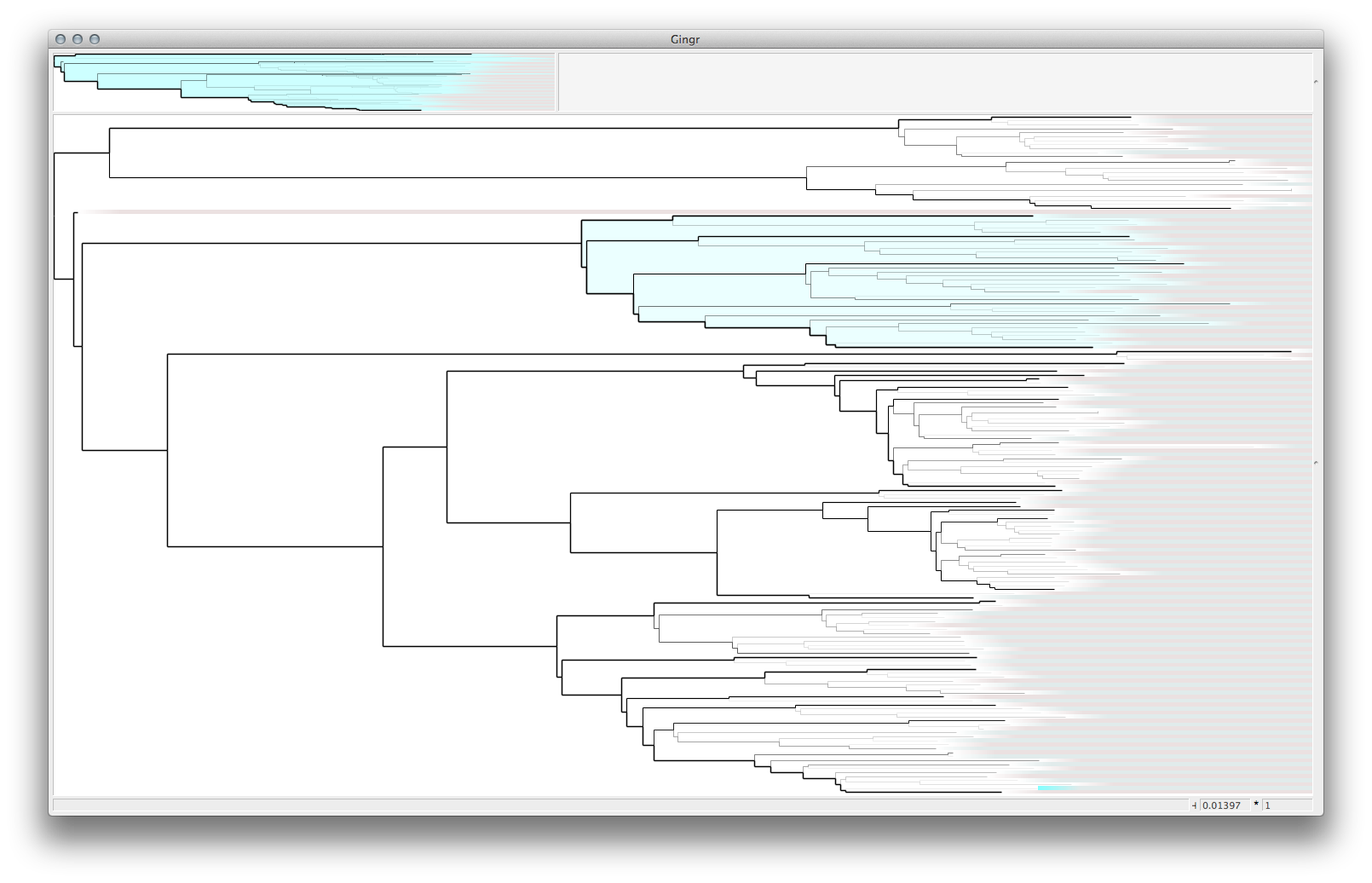 | 1127 (8%) |
| Parsnp | 8 (<1%) | 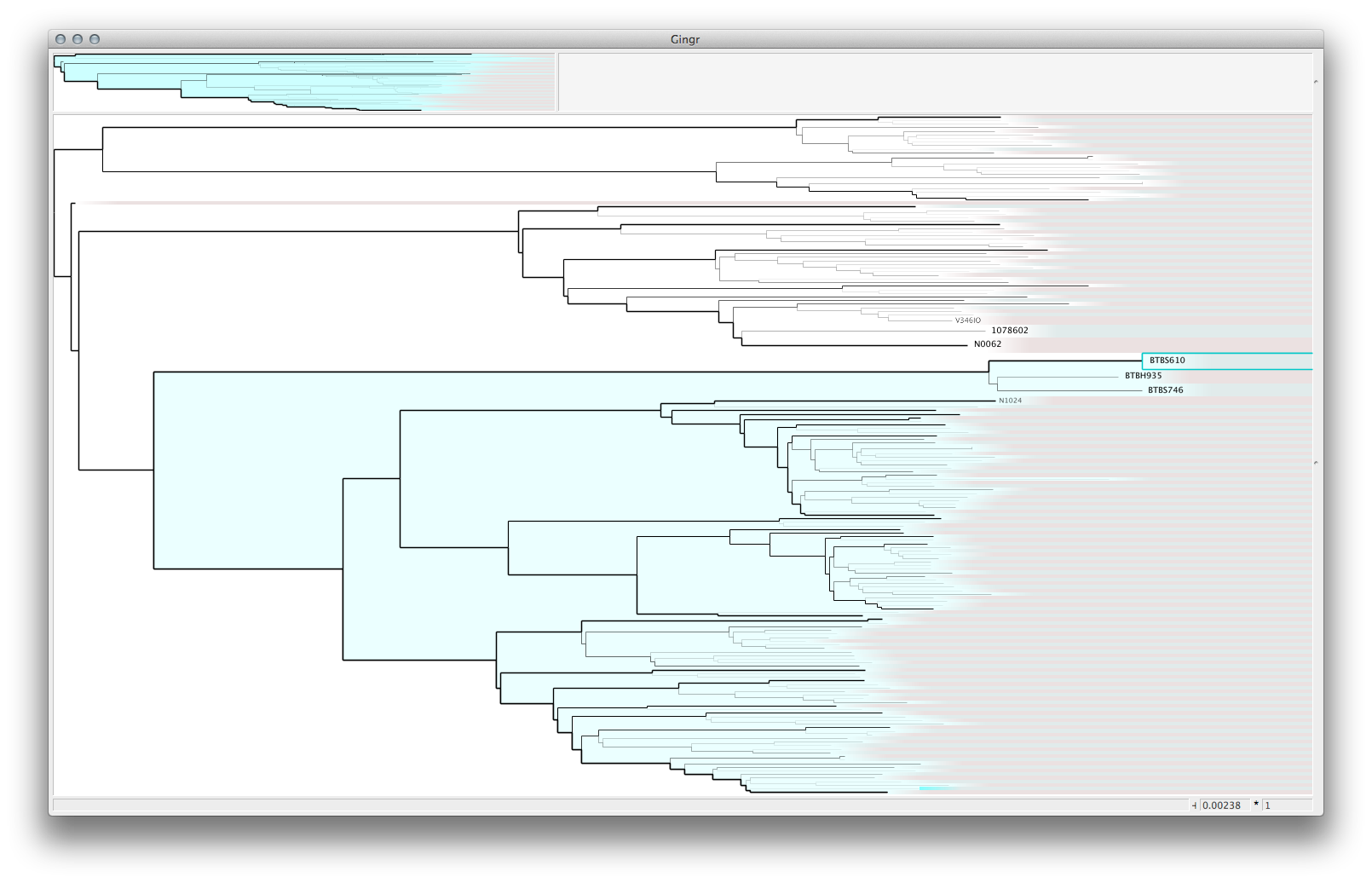 | 896 (6%) |
| Comas | 30 (1%) | 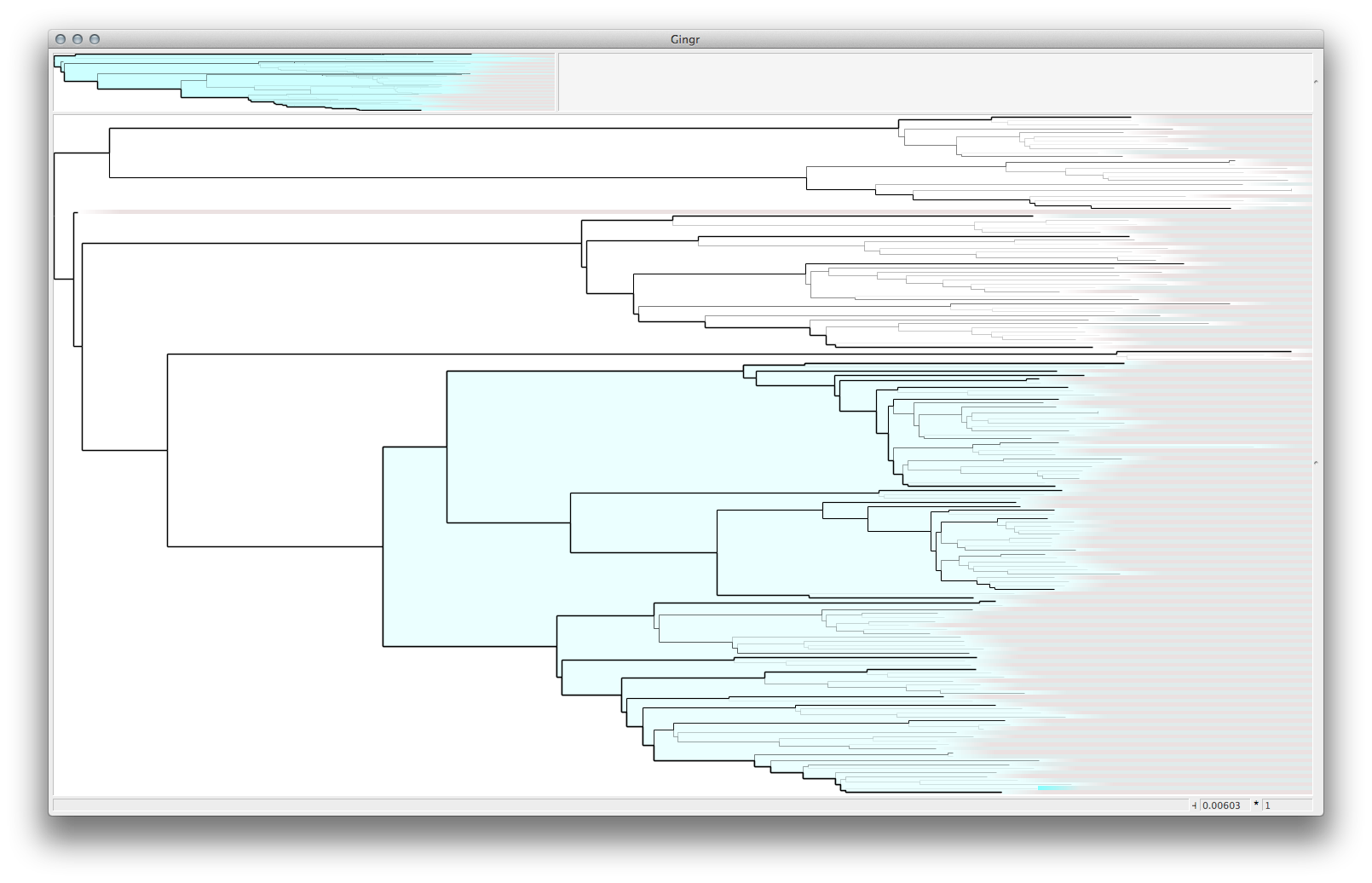 | 3270 (14%) |
| Comas | 3016 (63%) | Single genome splits | 3016 (13%) |
| Comas | 74 (2%) | 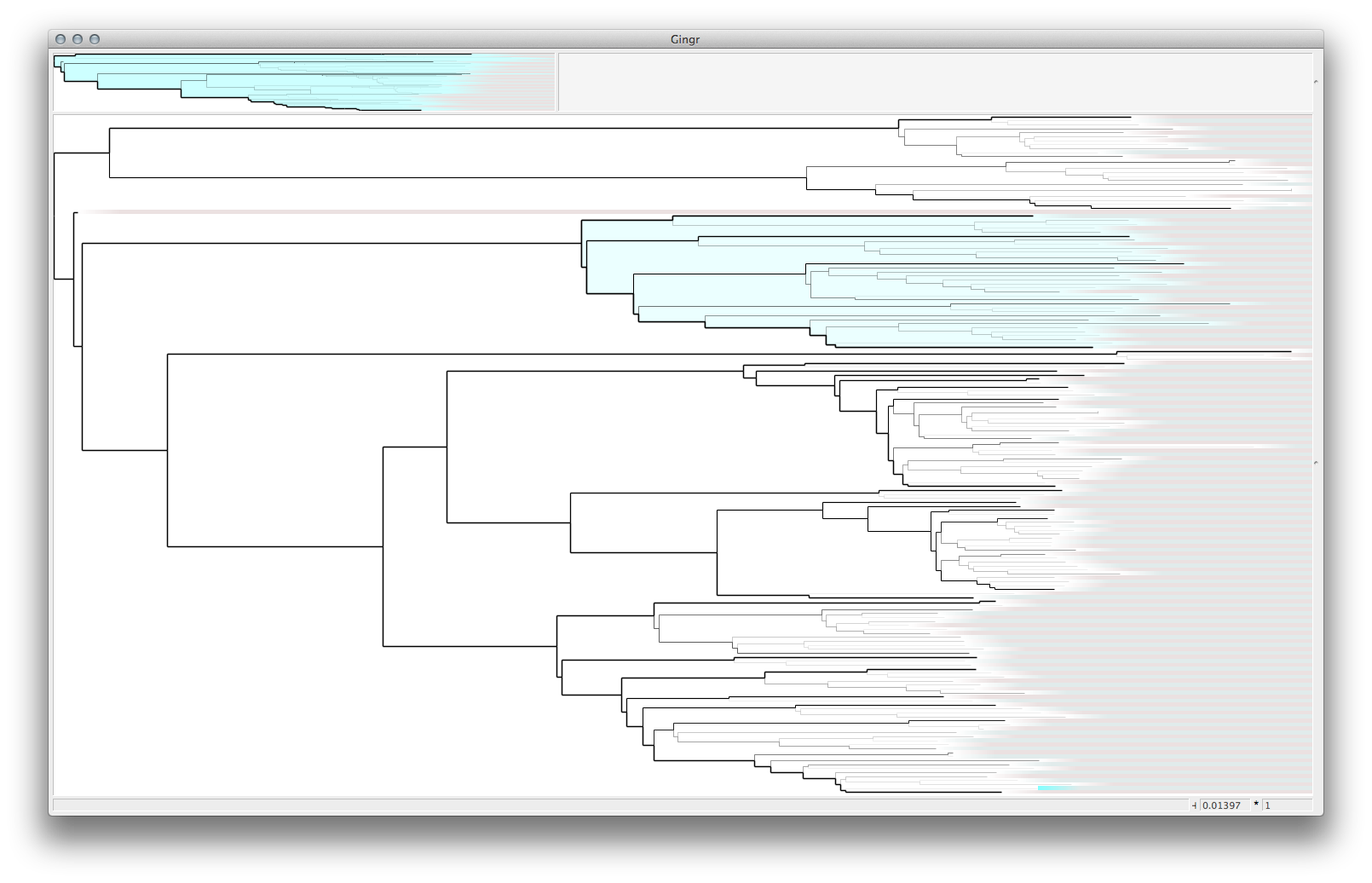 | 2516 (11%) |
| Comas | 109 (2%) | 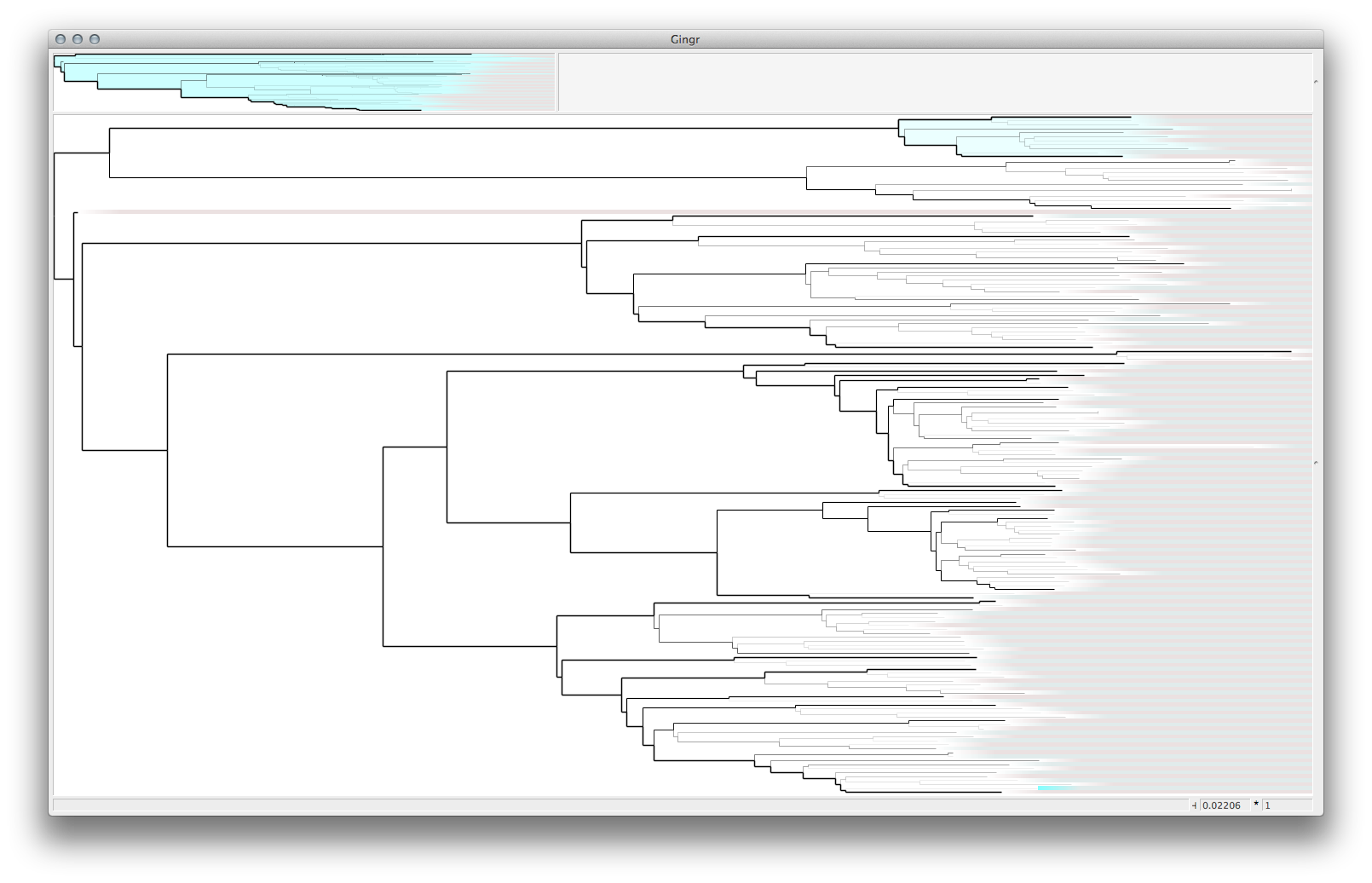 | 1199 (5%) |
| Comas *et al* | 32 (1%) | 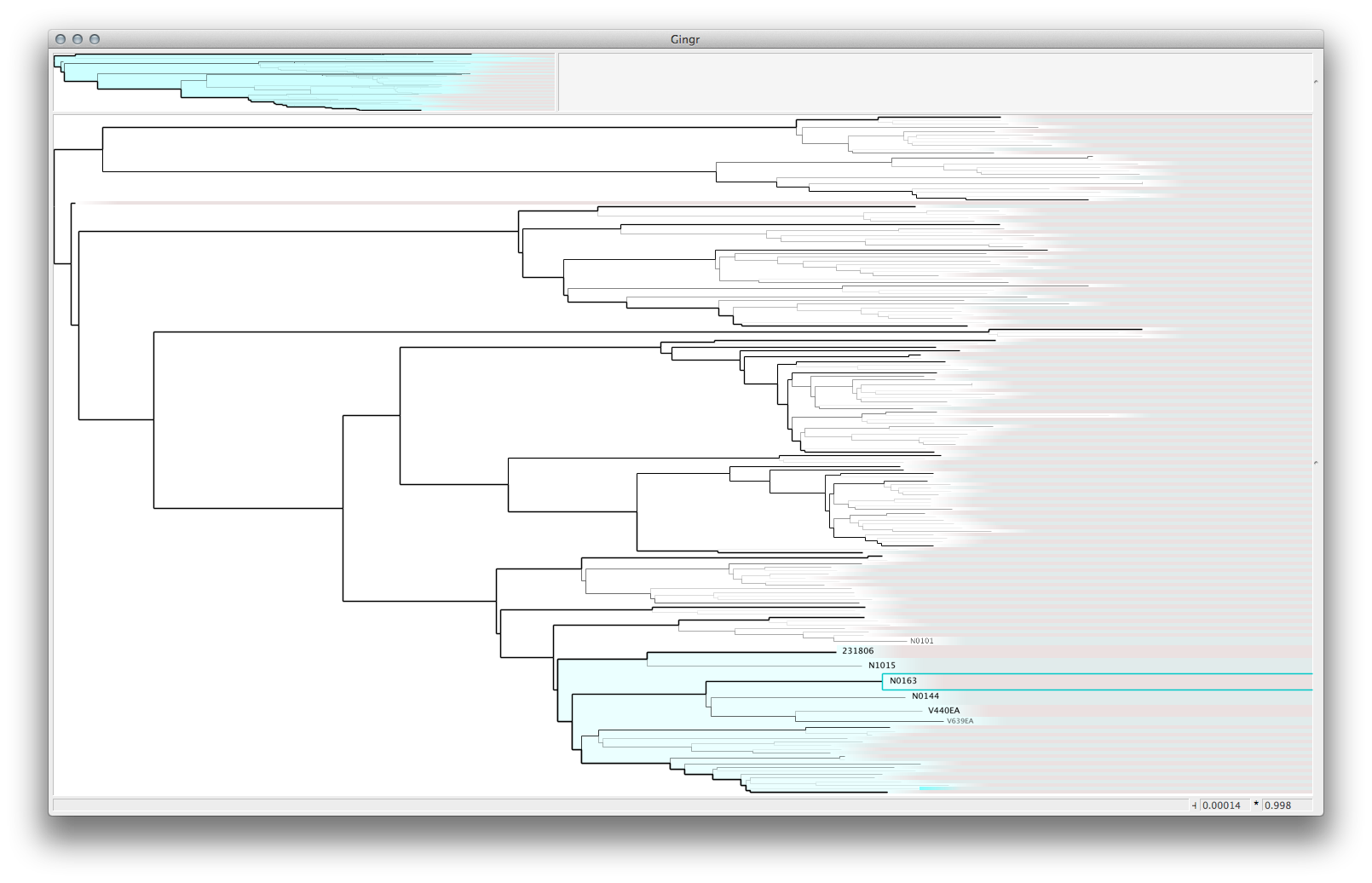 | 1127 (5%) |

**Supplementary Figure 1**. Runtime comparison for the whole-genome alignment methods on the simulated 32-genome *E. coli* W3110 dataset. The y-axis is log scale. pMauve = progressiveMauve, and Mugsy(p) indicates Mugsy with a parallelized NUCmer search. All programs were allocated 32 cores on the hardware noted above. Note that Mugsy is not multithreaded.

##

CPU runtime (minutes)

Number of genomes

**Supplementary Figure 2.** Timing performance from 32 to 10,000 *S. pneumoniae* genomes. The x-axis indicates the number of genomes and the y-axis the wall clock time for core-genome alignment. Alignments were performed on the hardware noted above. The gray line represents a linear time relationship between the number of genomes and search time.
